# Supplementary material for: Inkjet Printable and Self-Curable Disperse Dyes/P(St-BA-MAA) Nanosphere Inks for Both Hydrophilic and Hydrophobic Fabrics
Source: Polymers (Basel). 2018 Dec 18;10(12):1402. doi: 10.3390/polym10121402 (PMC6401999; doi:10.3390/polym10121402)
Supplement: Supplementary file 1 [file polymers-10-01402-s001.pdf]

Inkjet Printable and Self-Curable Disperse Dyes/P(St-BA-MAA) Nanosphere Inks for Both Hydrophilic and Hydrophobic Fabrics

Yawei Song [1,2,3], Kuanjun Fang [1,2,3,*], Yanfei Ren [1,2,3], Zhiyuan Tang [1,2,3], Rongqing Wang [1,2,3], Weichao Chen [1,2,3], Ruyi Xie [1,2,3], Zhen Shi [1,2,3] and Longyun Hao [1,2,3]

1 Fiber Materials and Modern Textiles of the Growing Base for State Key Laboratory, Qingdao University, 308 Ningxia Road, Qingdao 266071, China; 15864737168@163.com (Y.S.); tjpuryf@126.com (Y.R.) 18363995943@163.com (Z.T.); 17864283938@163.com (R.W.); chenwc@qdu.edu.cn (W.C.); xry1228@126.com (R.X.); shizhen988@126.com (Z.S.); hly1978@163.com (L.H.)

2 School of Textiles & Clothing, Qingdao University, 308 Ningxia Road, Qingdao 266071, China

3 Collaborative Innovation Center for Eco-Textiles of Shandong Province, 308 Ningxia Road, Qingdao 266071, China

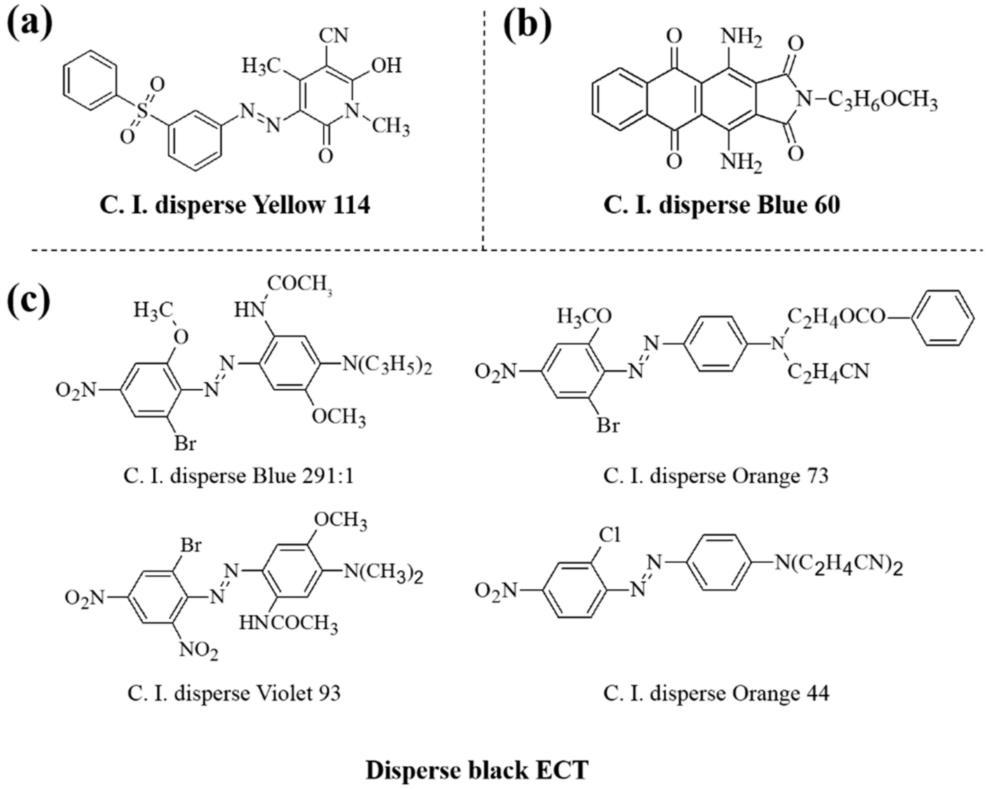

Scheme S1. Molecular structure of C. I. disperse Yellow 114 (**a**), C. I. disperse Blue 60 (**b**) and disperse black ECT (**c**) which combined with C. I. disperse Blue 291:1, C. I. disperse Orange 73, C. I. disperse Violet 93, C. I. disperse Orange 44.

Table S1. The CIE Lab color values of printed fabrics. [a]

Printed fabrics [b]	L*	a*	b*	c*	h°
Cotton-Red	68.66	36.43	11.32	38.15	17.26
Cotton-Blue	55.32	−10.16	−43.75	44.91	256.90
Cotton-Yellow	85.78	1.07	76.69	76.70	89.20
Cotton-Black	29.78	−0.12	0.29	0.31	111.60
Polyester-Red	71.61	36.18	9.06	37.30	14.06
Polyester-Blue	54.69	−8.71	−44.26	45.11	258.90
Polyester-Yellow	87.86	−3.68	78.24	78.33	92.70
Polyester-Black	28.08	−0.19	0.25	0.32	127.70

[a.] Printing parameters: Pixel 500 dpi, 5 pass. [b.] All the fabrics were modified by EPTAC and baked at 150 °C for 2 min.
